# Functional Characterization of the Rice UDP-glucose 4-epimerase 1, *OsUGE1*: A Potential Role in Cell Wall Carbohydrate Partitioning during Limiting Nitrogen Conditions

**DOI:** 10.1371/journal.pone.0096158

**Published:** 2014-05-01

**Authors:** David R. Guevara, Ashraf El-Kereamy, Mahmoud W. Yaish, Yong Mei-Bi, Steven J. Rothstein

**Affiliations:** 1 Department of Biology, College of Science, Sultan Qaboos University, Muscat, Oman; 2 Department of Molecular and Cellular Biology, University of Guelph, Guelph, Ontario, Canada; Lawrence Berkeley National Laboratory, United States of America

## Abstract

Plants grown under inadequate mineralized nitrogen (N) levels undergo N and carbon (C) metabolic re-programming which leads to significant changes in both soluble and insoluble carbohydrate profiles. However, relatively little information is available on the genetic factors controlling carbohydrate partitioning during adaptation to N-limitation conditions in plants. A gene encoding a uridine-diphospho-(UDP)-glucose 4-epimerase (OsUGE-1) from rice (*Oryza sativa*) was found to be N-responsive. We developed transgenic rice plants to constitutively over-express the *OsUGE-1* gene (OsUGE1-OX1–2). The transgenic rice lines were similar in size to wild-type plants at the vegetative stage and at maturity regardless of the N-level tested. However, OsUGE1-OX lines maintained 18–24% more sucrose and 12–22% less cellulose in shoots compared to wild-type when subjected to sub-optimal N-levels. Interestingly, OsUGE1-OX lines maintained proportionally more galactose and glucose in the hemicellulosic polysaccharide profile of plants compared to wild-type plants when grown under low N. The altered cell wall C-partitioning during N-limitation in the OsUGE1-OX lines appears to be mediated by OsUGE1 via the repression of the cellulose synthesis associated genes, *OsSus1*, *OsCesA4*, *7*, and *9.* This relationship may implicate a novel control point for the deposition of UDP-glucose to the complex polysaccharide profiles of rice cell walls. However, a direct relationship between OsUGE1 and cell wall C-partitioning during N-limitation requires further investigation.

## Introduction

Nitrogen (N) is a major mineral nutrient required for the biosynthesis of molecules such as amino acids, chlorophyll, nucleic acids, and proteins that are vital components for plant growth and development. Generally, soil N deficiency leads to decreased plant growth and productivity, and thus insufficient N levels in agricultural areas severely limit crop yield potential. While the application of N fertilizer in the form of nitrate is essential for achieving maximum crop yields, the unused nitrate that is applied in agricultural areas is a major source of environmental N pollution [1]. Therefore, a major goal in plant research is to develop crop plants that display improved N use efficiency (NUE) to attain higher yields and limit N fertilizer-based environmental pollution.

N-limitation has placed selective pressure for the acquisition of traits that allow plants to complete their life cycle under sub-optimal N levels, and has greatly influenced plant genome evolution [2]. Traits enabling plants to acclimate to N-limitation include a reduction in growth rate and photosynthesis, N remobilization from older to younger leaves, and anthocyanin, starch and sugar accumulation [3]. Over the past decade, investigation on the molecular mechanisms enabling plant adaptation to N-limitation has been pursued using whole-genome transcript profiling of plants grown under sub-optimal N-levels. This work has uncovered that plant responses to N-limitation are complex, as hundreds of genes have been found to be N-responsive or regulated by N [4]–[6]. This complexity is further confounded by the finding that significant interplay exists between N and carbon (C) metabolism during adaptation to low N environments at the level of the transcriptome [4]–[8] and metabolome [9]. Thus, delineating the genetic basis enabling plants to adapt to N-limitation has been a considerable challenge due, in part, to the significant diversity in the genes implicated in playing an adaptive role during N-limitation adaptation.

A positive correlation between photosynthetic rate and leaf total N content has been well established [10]. Lower rates of photosynthesis during growth under N-limitation are attributed to a reduction in chlorophyll content and Rubisco activity [10], [11]. Also, hexose:amino acid ratios appear to be important for photosynthetic repression during N-limitation [12]. Furthermore, C-compounds and amino acids have been found to reciprocally modulate the expression of key genes involved in N-assimilation [13]. Taken together, these observations reveal that C-partitioning must be tightly regulated in an N-dependent manner to ensure that an adequate supply of C-skeletons precursors is available for N-assimilation and for the synthesis of complex carbohydrates such as starch and cellulose under conditions of lower C-gain where C is limiting. Unfortunately, relatively little information is available on the genetic factors controlling C partitioning during N-limitation conditions. Thus, a greater understanding of the complex C x N interactions is required in order to engineer plants that are capable of attaining higher biomass and yield when grown on soils containing sub-optimal levels of N [14], [15].

Based on microarray analyses, we identified several genes encoding uridine 5′-diphospho-glucose 4-epimerases (UGEs) from rice and *Arabidopsis* [4], [16] that have been found to be N-responsive, with specific AtUGEs being more prominently expressed in an N-dependent manner [4], [16]. The *Arabidopsis* genome encodes a family of five UGE isoforms, and all *uge* single mutants show normal shoot and root growth and fecundity, while mutant combinations revealed an overlap and synergy in function between the different isoforms rather than isolated and distinct functions [17]. Intriguingly, transgenic *Arabidopsis* over-expressing a rice UGE (*OsUGE-1*) were more tolerant than wild-type to drought, freezing temperatures, and high salinity due, in part, to elevated levels of raffinose [18]. Taken together, while UGEs are implicated in cell wall carbohydrate metabolism [17], [19] and abiotic stress tolerance [18], the importance of the rice *OsUGE1* for adaptation to low N has not been established. The rice gene, *OsUGE1*, previously identified as a strong N-responsive gene by our group, was constitutively over-expressed under the control of the maize ubiquitin promoter in rice. In this work, we report on the analysis of transgenic rice lines over-expressing the *OsUGE1* and show that *OsUGE1* plays an important role in cell wall C partitioning during adaptation to N-limiting conditions.

## Materials and Methods

### Plant Growth Conditions

*Oryza sativa Japonica* cv. Kaybonnet *OsUGE1* overexpression lines (OsUGEOX1–2) and respective wild-type controls plants were either grown on a peat moss and vermiculite (1∶4) soil medium (SunGro Horticulture Canada Ltd., BC, Canada) or under hydroponic conditions, supplemented with a nutrient solution containing: 4 mM MgSO_4_, 5 mM KCl, 5 mM CaCl_2_, 1 mM KH_2_PO_4_, 0.1 mM Fe-EDTA, 0.5 mM MES (pH 6.0), 9 µM MnSO4, 0.7 µM Zn SO_4_, 0.3 µM CuSO_4_, 46 µM NaB_4_O_7_ and 0.2 µM (NH4) 6Mo_7_O_2_. 10 mM or 3 mM nitrate was used as the “high N” treatment in soil or hydroponic conditions, respectively, while 3 mM or 1 mM nitrate was used as the “low N” treatment under soil or hydroponic conditions, respectively. Plants were grown in a growth room with 16 hour light (∼500 µmolm^−2^s^−1^) at 29°C and 8 hour dark at 23°C for four weeks. Shoots and roots were harvested separately in liquid nitrogen and stored at −80°C until further analysis.

### Transgenic Rice Plants

The constructs for over-expressing *OsUGE1* were made using the maize ubiquitin promoter. The full length cDNA of *OsUGE1* was amplified and cloned into a binary vector. Transgenic rice plants were generated through Agrobacterium-mediated transformation to constitutively over-express the *OsUGE1* gene [20]. For segregation and molecular analysis of transgenic plants, plants were grown on a peat moss and vermiculite (1∶4) soil medium (SunGro Horticulture Canada Ltd., BC, Canada) a slow-release fertilizer, Nutricote total 13-13-13 (Plant Products Co. Ltd., ON, Canada) was used as the nutrient source for plants. Phosphomannose isomerase (PMI) tests were used for genotyping to detect the selectable marker PMI [21]. PMI testing for the ten independent events generated revealed that half of the events displayed a single chromosomal insertion site of the candidate gene, as genotyping results showed a 3∶1 segregation ratio of these events and responded similarly to N treatments (data not shown). Two transgenic lines were selected for metabolic profiling of C and N metabolites.

### Sugar Feeding Experiments

Individual seeds were placed in wells containing 1 mL of 55 mM a monosaccharide sugars solubilized in water in 24-well plates, and kept in an incubator in the dark at 25°C for 96 hours. The coleoptile was measured after the incubation period with or without sugar exposure.

### Analysis of Recombinant OsUGE1

The coding sequence of the OsUGE-1 was amplified using PCR and cloned into the pET-15b plasmid vector (Novagen). The resulting plasmid was transformed into *E. coli* BL21 (DE3) and grown in LB medium, and IPTG (isopropyl-1-thio-β-D-galactopyranoside) was used to induce the expression of the recombinant protein [22]. The enzyme was purified from the cell lysate using a Ni^2+^-NTA affinity column that binds to the epimerase’s N-terminal hexahistidine sequence [22]. The enzyme activity assays were performed as outlined in Chen et al. (1999)[22]. Briefly, OsUGE1 activity was assayed in a 200 µL reaction mixture containing 20 mM Tris/HCl (pH 8.0), 1 mM NAD^+^, and 1 mM UDP-gal or UDP-glc, and incubated at 37°C for 30 minutes, and quickly terminated by transferring the reaction tube to 100°C for 5 minutes. The UDP-gal or UDP-glc products were quantified using capillary electrophoresis using a P/ACE MDQ Glycoprotein System (Beckman Coulter) with UV detection, according to Westman et al. (2006)[23].

### Metabolite Analysis

Rice seedling tissues were extracted and analyzed using the previously reported protocol [24]. Briefly, 200 mg FW of tissue was extracted with 1 mL 100% methanol with shaking at 70°C for 15 min. Ribitol was added as an internal standard to the samples during extraction, and extracts were separated into polar (methanol/water) and apolar/lipid (chloroform) phases. Polar fractions were vacuum dried and derivatized using methoxyamine and N-methyl-N-trimethysilyl-trifluoroacetamide. The derivatized samples were diluted 5-fold, and then 1 µL was injected into the splitless injection port of a Varian 1200 gas chromatography-mass spectrometry system (Varian Inc. CA, USA). Chromatography was performed using a 30-m×0.25-mm Rtx-5 MS column (Chromatographic Specialties, ON, Canada). For each run, the gas chromatograph oven was set at 70°C initial temperature, which was held for 5 min, and increased at 5°C min–1 to 310°C, at which it was held for 6 min. The injection temperature was 230°C, and the ion source was kept at 200°C. Mass spectra were recorded at three scans per second with an m/z scanning range of 50 to 650. The data analysis was performed using the automated mass spectral deconvolution and identification system (AMDIS) software (http://chemdata.nist.gov/mass-spc/amdis).

### Galactose, Glucose, Sucrose, RFO, and Starch Quantification

Polar metabolites were extracted from 200 mg FW of leaf tissue or 10 mg DW of fine-powdered aliquot from 100 seeds at 70°C using 3×1 mL 100% HPLC grade methanol. Phase separation of the pooled methanolic extractions was carried out using methanol:chloroform:water (5∶3∶7, v/v/v) and the polar phase was removed, lyophilized and resuspended in water to quantify soluble sugars. Briefly, glucose was quantified by measuring the ΔNADPH concentration at 340 nm following the addition of hexokinase/glucose-6-phosphate dehydrogenase, and the sample was treated with invertase to quantify sucrose-based glucose moieties using the same method using a glucose/sucrose quantification kit (Megazyme, Ireland) as specified by the manufacturer. Galactose and raffinose were quantified using a galactose/raffinose quantification kit (Megazyme, Ireland) as specified by the manufacturer. The remaining insoluble pellet after methanol extractions was lyophilized and suspended in water and heated to 100°C for 30 minutes. Total starch was determined using a starch quantification kit (Megazyme, Ireland) as specified by the manufacturer.

### Cell Wall Extraction and Analysis

Hemicellulosic sugars were hydrolyzed using 2 M trifluoroacetic acid and quantified as reported in Rösti et al. (2007) [17]. Briefly, following the removal of starch from the insoluble pellet described above, ∼2 mg DW of the insoluble pellet was mixed with 200 µL of water containing 50 µg of myo-inositol as an internal standard. To the suspension, 200 µL of 4 M trifluoroacetic acid was added and the hemicellulosic sugars were hydrolyzed at 121°C for 1 hour. The hydrolyzed sample was removed and mixed with 300 µL of HPLC grade isopropanol, and the extract was dried at 45°C. The dried extract was re-suspended in 400 µL of water into a GC vial, lyophilized and the monosaccharides were derivatized using methoxyamine/N-methyl-N-trimethysilyl trifluoroacetamide and quantified using GC/MS, following the same method described. After the removal of the hemicellulosic sugars, cellulose was determined by quantifying the hydrolyzed glucose moieties by treating the remaining insoluble pellet with 2U of cellulase using the *Trichoderma viridae* cellulase digestion for 24 hours, as previously described [25], and quantifying the glucose moieties from the cellulose digestion using the glucose oxidase/peroxidase (GOPOD) method quantification kit (Megazyme, Ireland) following the specifications by the manufacturer.

### Real-time PCR Analysis

Total RNA was isolated from plant tissues using TRI-Reagent (Sigma-Aldrich, MO, USA). To eliminate any residual genomic DNA, total RNA was treated with RQ1 RNase-free DNase (Promega, WI, USA). The first strand cDNA was synthesized from total RNA by using the Reverse Transcription System kit (Quanta, MD, USA). Primer Express 2.0 software (Applied Biosystems, CA, USA) was used to design the primers for the target genes (*OsUGE1F*, tggtgttctcatcatccgca; *OsUGE1R*, cgattaccagctttgttctgcc:

*SUS1F*, catctcaggctgagactctga; *SUS1R*, caaattcaatcgaccttactt: *CesA4F*, gagaccaccaccaccaacag; *CesA4R*, gacaaagcccagagagaggaaa: *CesA7F*, ttcatccccaagcccaag;

*CesA7R*, caagaatcatccatccggtca: *CesA9F*, actgctgagagagggcgtca; *CesA9R*, aacatagcacgaactcaacacga: *Actin2F*, tcttacggaggctccacttaac; *Actin2R*, tccactagcatagagggaaagc). Relative quantification (RQ) values for each target gene were calculated by the 2^−ΔΔC^_Τ_ method [26] where the expression levels were normalized using the house keeping *Actin2* gene.

## Results

### Expression Profiles of UGEs in Rice Support Distinct *in planta* Roles Throughout Development

Uridine 5′-diphospho-glucose 4-epimerase (UGE; EC 5.1.3.2) catalyzes the inter-conversion of UDP-glucose (UDP-glc) and UDP-galactose (UDP-gal) which are important precursors for the biosynthesis of soluble sugars such as sucrose, and the important precursor for raffinose family oligosaccharides (RFO), galactinol, respectively or for the synthesis of macromolecular components of the cell wall such as cellulose or hemicellulose. The rice genome contains four putative UGE-encoding genes (OsUGE1–4). All four predicted OsUGEs in rice carry an epimerase domain and belong to the NAD^+^ dependent epimerase/dehydratase family proteins that use NAD^+^ as a cofactor and nucleotide-sugars as substrates **(Figure S1)**. This apparent redundancy of UGEs in rice is not unique as three and five isoforms of UGEs have been identified and characterized in barley (*Hordeum vulgare* L., HvUGE1–3; [27]) and *Arabidopsis* (*Arabidopsis thaliana*, AtUGE1–5; [28]), respectively. Several studies have shown that different UGE isoforms have a broad range of metabolic roles *in vivo* including the synthesis of polysaccharides and glycoproteins to support the cell wall, and are also important for carbohydrate catabolism [27], [28].

**Figure 1:**
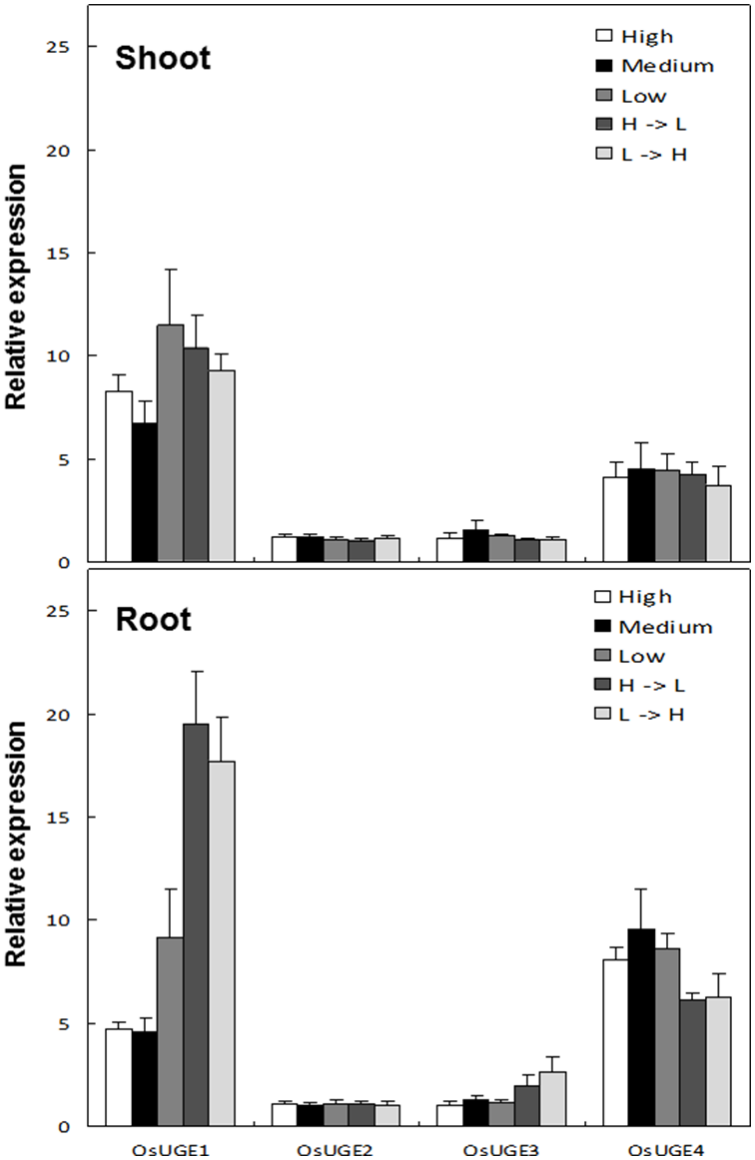
Expression pattern of *OsUGE*s in shoots or roots from rice plants subjected to N-treatments. Low (L) = 1 mM, Medium (M) = 3 mM, High (H) = 10 mM nitrate, mean±SD, n = 3. H = >L and L = >H indicate the treatment of the plant with high followed by low and low followed by high nitrate concentrations, respectively.

Whole-genome transcript profiles of tissues harvested from rice plants subjected to N-treatments were examined to identify genes involved in N-limitation adaptation according the previously described method [29]. The results showed that of the four encoded UGEs in rice, *OsUGE1* (LOC_Os05g51670) was the only transcript present at higher levels in the roots of rice plants subjected to N-induction or N-reduction experiments, and in plants grown continuously at low (1 mM) nitrate compared to plants grown under high (10 mM) nitrate concentrations (**Figure 1**). None of the *OsUGE*s significantly changed in expression in rice shoots from plants subjected to the different N-treatments (**Figure 1**). Thus, *OsUGE1* appears to be the only root N-responsive *UGE* in rice.

### Analysis of Rice Lines Over-expressing *OsUGE1*

**Figure 2:**
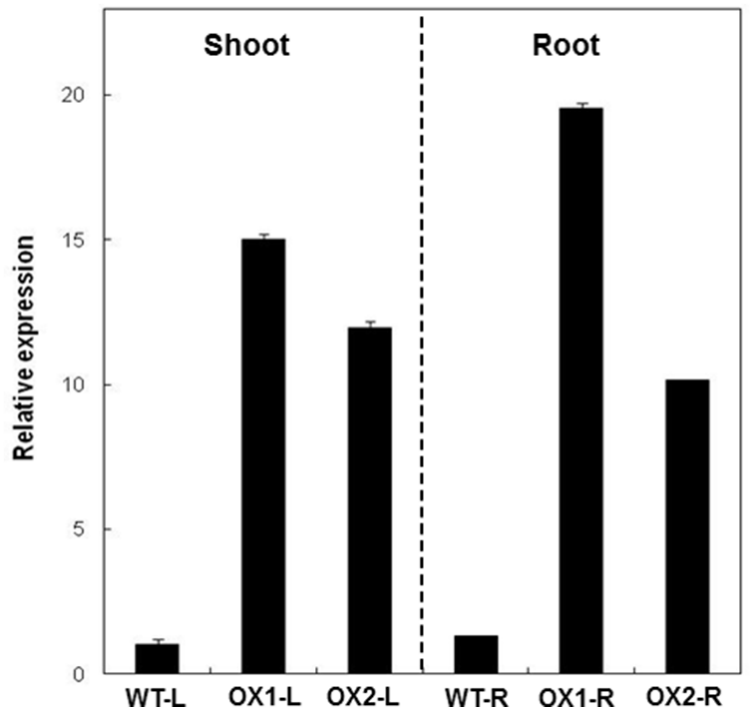
Level of *OsUGE1* over-expression in shoots and roots of OsUGE1-OX lines.

Transgenic OsUGE1-OX rice plants displayed an 11–15-fold and 10–18-fold higher level of *OsUGE1* expression in the shoots and roots, respectively (**Figure 2**). To test the response of the OsUGE1-OX lines to N-limitation, plants were grown under the defined limiting N (1 mM nitrate) or high N (3 mM nitrate) growth conditions, as described in Bi et al. (2009) [29]. Transgenic rice plants over-expressing the *OsUGE1* gene showed no obvious difference in shoot or root dry biomass compared to wild-type plants at four weeks after germination, and achieved similar biomass and seed yield at maturity, relative to wild-type plants when grown under N-limiting or high N environmental conditions. Therefore, the shoot portion of the OsUGE1-OX lines and respective wild-type controls were characterized to determine the impact of *OsUGE1* overexpression on carbohydrate metabolism in response to different N-treatments.

**Figure 3:**
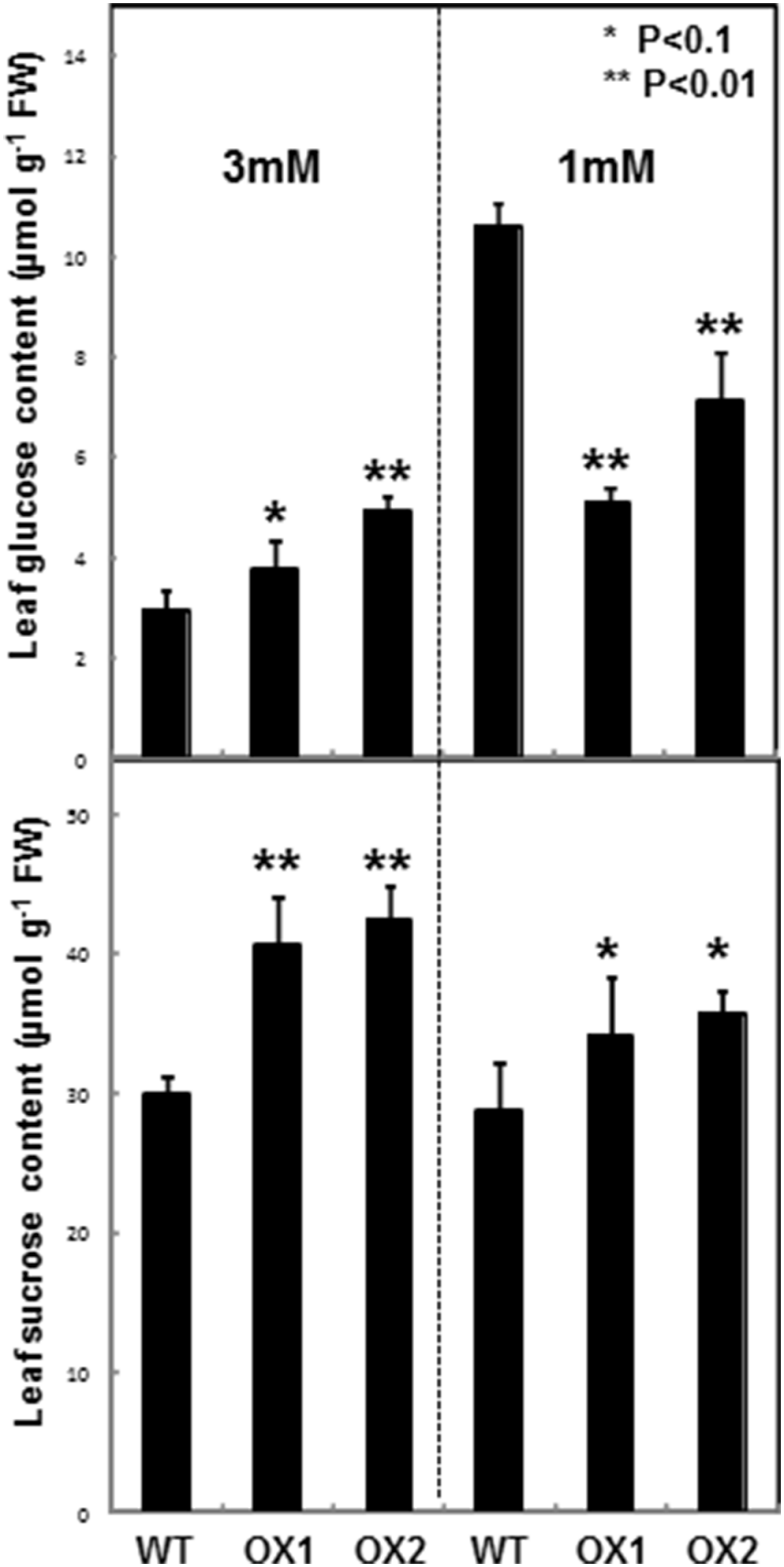
Leaf glucose and sucrose content of four week old rice plants subjected to ample (3 mM) or limiting (1 mM) nitrate conditions. Mean±SD, n = 6.

In wild-type rice plant, OsUGE1 is induced by nitrogen limitation conditions and has a potential role in C partitioning. In order to identify the molecular basis behind N- response and its relation with C partitioning, we performed GC-MS based metabolic profiling analysis of OsUGE1-OX lines and wild-type plants when both are subjected to ample or N-limitation conditions. We found that the levels of only glucose and sucrose were significantly altered in OsUGE1-OX line compared to wild-type plants grown under N-limitation conditions (**Figure 3**). For example, OsUGE1-OX line maintained higher sucrose contents in leaves of plants grown at low (18 to 24%) or high (36 to 42%) N compared to wild-type. Interestingly, while OsUGE1-OX line had 27 to 68% more glucose compared to wild-type plants grown under ample N, OsUGE1-OX line had 1.5 to 2-fold lower glucose levels when grown under sub-optimal N-levels compared to wild-type. Based on the analysis of a limited number of metabolic profiles, a few metabolites are changing in the UGEOX lines under limiting nitrogen conditions including threonine, galactinol and raffinose however, the tested metabolic profiles were not significantly different (*p*≤0.05) between OsUGE1-OX lines and wild-type plants subjected to ample or N-limiting conditions (Table S1). These similar but limited metabolic profiles do not necessarily disprove the notion that other unidentified metabolites are differentially accumulated in OsUGE1-OX lines and wild-type plants subjected to ample or N-limiting conditions. These findings are in contrast to work reported by Liu et al. (2007) [18] which demonstrated that OsUGE1 over-expression in *Arabidopsis* led to an accumulation of RFOs in leaves when plants were grown under normal conditions. Interestingly, the seeds of the transgenic plants had 9–13% more sucrose, with a concomitant 4–11% decrease in RFOs compared to wild-type seeds, and no differences in galactose or glucose, and starch contents were observed **(** **Table 1** **)**. Taken together, the higher sucrose and lower glucose contents displayed in shoots of OsUGE1-OX lines compared to wild-type plants indicates that OsUGE1 could be important for influencing sucrose biosynthesis during growth under N-limitation conditions.

**Table 1:** Seed characteristics of rice plants over-expressing *OsUGE1* compared to wild-type rice.

Measurement	WT	OsUGE1-OX1	OsUGE1-OX2
Total carbon (% DW)	44.0±0.3	43.8±0.4	44.0±0.8
Total protein (% DW)	10.0±0.5	10.2±0.1	10.4±0.8
Starch content (mg.g^−1^ DW)	798.6±53.4	805.9±47.5	793.2±50.3
Sucrose (mg.g^−1^ DW)	5.4±0.4	6.1±0.3*	5.9±0.1*
Total RFO (µg.g^−1^ DW)	89.5±7.9	80.7±4.9*	85.7±1.5*
Galactose (µg.g^−1^ DW)	33.3±4.2	35.0±5.4	34.7±2.3
Glucose (µg.g^−1^ DW)	107±9.6	138±48	151±44

Mean ± SE, n = 3, P<0.05.

### OsUGE1 Enzyme has Higher Affinity for UDP-gal *in* *vitro*

**Figure 4:**
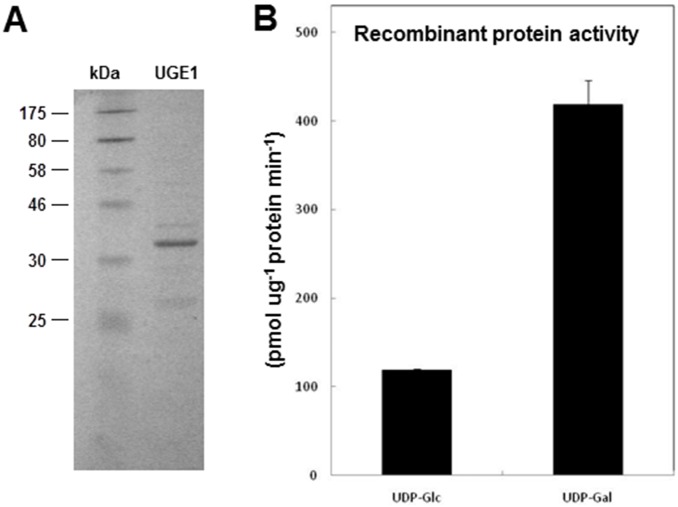
Analysis of recombinant OsUGE1 in expressed and purified from *Escherichia coli*. A) SDS/PAGE separation of OsUGE1. Protein molecular mass standards (in KDa) are indicated on the left. B) UDP-Gal and UDP-Glc substrate specificity of recombinant purified OsUGE1. Means ± SD, n = 3.

UGEs interconvert UDP-glucose (UDP-glc) and UDP-galactose (UDP-gal) [17], [28]. In order to study the enzymatic activity of OsUGE1 against UDP-glc and UDP-gal *in vitro,* and to test whether OsUGE1 is more prominently influences C-flux in the sucrose biosynthesis direction, the OsUGE1 recombinant protein was produced in *E. coli* and purified using the histidine tag fusion system. SDS-PAGE analysis revealed that the encoded OsUGE1 isoform is approximately ∼39 kDA (**Figure 4A**) consistent with the predicted weight of the encoded 354 amino acid residue single polypeptide (www.tigr.org). The recombinant OsUGE1 exhibited the highest activity in the conversion of UDP-gal into UDP-glc (**Figure 4B**). Although it was able to utilize both UDP-glc and UDP-gal as substrates, the recombinant OsUGE1 showed a ratio of ∼3∶1 UDP-glc and UDP-gal at equilibrium. These findings are consistent with work performed by Kim et al. (2009) [30] which determined the k_cat_/k_m_ for all OsUGEs and revealed that OsUGE1 most efficiently converts UDP-gal into UDP-glc compared to the other OsUGEs.

### Cell Wall Carbohydrate Analysis of OsUGE1-OX Lines

Phylogenetic analysis of the UGEs from rice and *Arabidopsis* revealed that the OsUGE1 most closely resembles the AtUGE’s 2, 4 & 5 isoforms [17], [18]. The AtUGE 2 & 4 isoforms synergistically influence shoot and root growth by modifying the cell wall carbohydrates in *Arabidopsis* [17]. Given the close association of *OsUGE1* to *Arabidopsis* UGE isoforms associated with cell wall carbohydrate deposition, we examined both the hemicellulosic and cellulosic polysaccharide profiles of leaves of four week old OsUGE1-OX lines and wild-type subjected to ample (3 mM) or N-limiting (1 mM) conditions.

**Figure 5:**
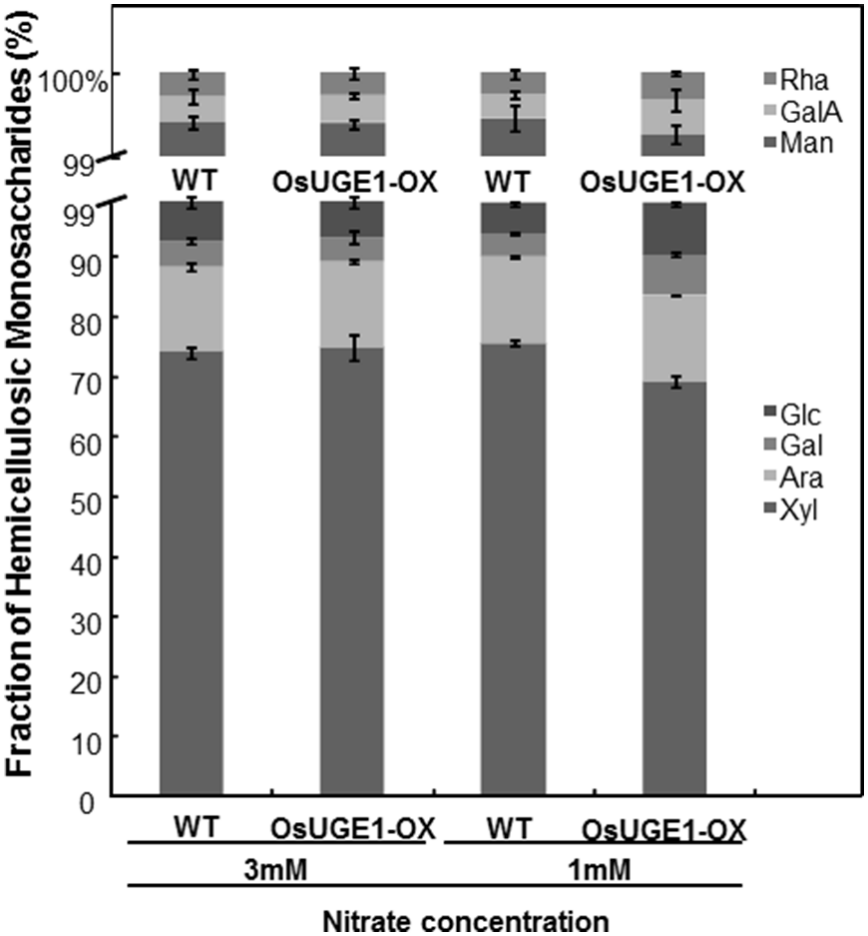
Hemicellulose profile of leaves of rice plants subjected to ample (3 mM) or limiting (1 mM) nitrate conditions. Mean±SD, n = 3.

Hemicellulose is the second most abundant polymer in the plant cell wall after cellulose [31]. The hemicellulosic component of the cell wall consists of a β-1-4-linked polysaccharide composed of arabinose (ara), galactose (gal), glucose (glc), mannose (man), xylose (xyl), fucose (fuc), rhamnose (rha), and the sugar composition differs among different species [32]. Xyl was the most prominent hemicellulosic sugar followed by ara, and man, galA, and rha together accounted for only ∼1% of the total hemicellulosic sugar content (**Figure 5**). We did not detect fuc in all samples tested consistent with previous reports on rice shoot hemicelluloses analysis [33]. We found that hemicellulosic gal and glc together accounted for <10% of cell wall sugar content (**Figure 5**). No significant differences were observed in the hemicellulosic gal or glc contents between OsUGE1-OX lines and wild-type plants grown under ample N, however shoots contained proportionately more gal and glc in the hemicellulosic polysaccharide profile of plants exposed to low N at the expense of xyl (**Figure 5**).

**Figure 6:**
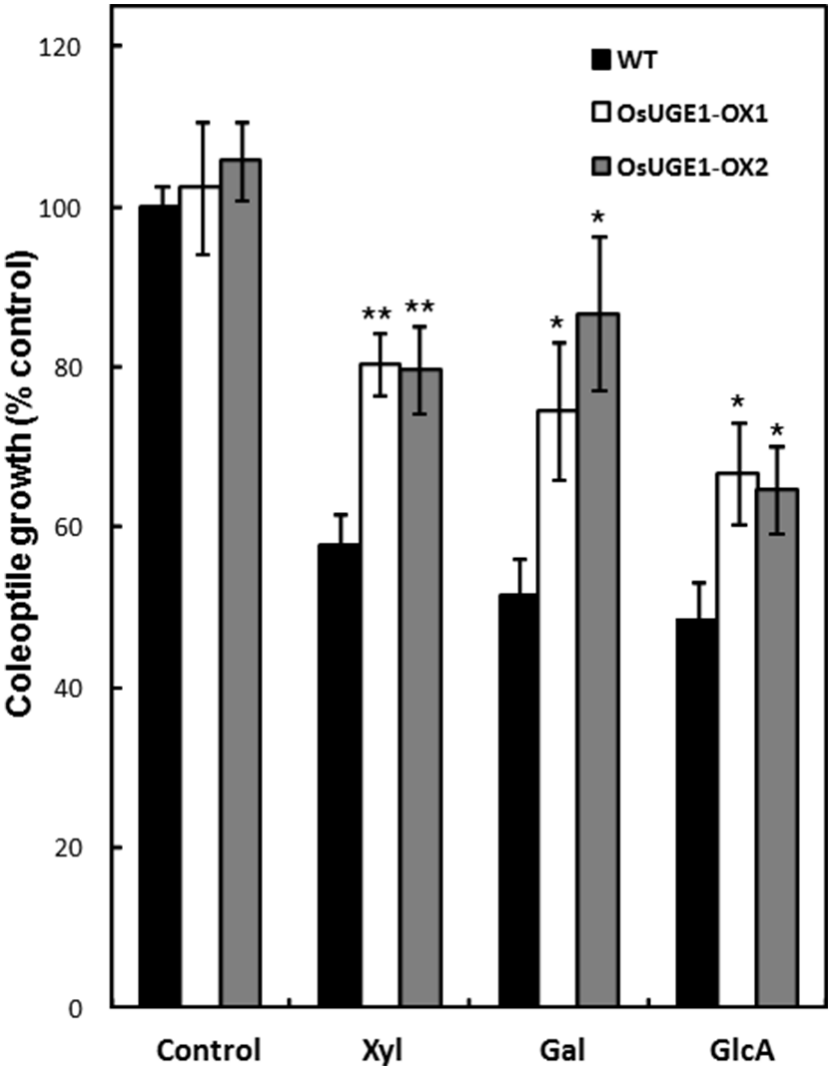
The effect of exogenously applied hexoses on dark-grown OsUGE1-OX and wild-type rice coleoptiles. Asterisks denoting on the figure when P<0.05 (*) or P<0.01 (**). Seeds were germinated in the presence of water (control) or 55 mM sugar in the dark, and the coleoptile length was measured after 96 hours. Mean±SD, n = 12, from two separate experiments.

To further test whether OsUGE1-OX lines can utilize sugars associated with the hemicellulosic polysaccharide profiles more efficiently than wild-type rice, seeds were treated with water, or 55 mM sugars, and coleoptile growth was measured according to the previously described method [34] (**Figure 6**). Both wild-type seeds and OsUGE1-OX lines achieved maximum coleoptile growth in the absence of sugar. Wild-type coleoptiles were inhibited in the presence of glc, rha, ara, and fuc compared to controls with a decrease of less than 25%. In the presence of xyl, gal, galA, and glcA the coleoptiles attained 40–50% the length of controls that received no exogenous monosaccharides **(Figure S2)**. Interestingly, OsUGE1-OX coleoptiles were 30–40% longer compared to wild-type in the presence of xyl, gal, and glcA (**Figure 6**), indicating that the transgenic lines had a superior ability to utilize these sugars as a carbon source for growth. The observation that OsUGE1-OX lines displayed much less growth inhibition in the presence of galactose provides further evidence that OsUGE1 influences the flux of C towards the generation of UDP-glc from UDP-gal *in vivo*.

**Figure 7:**
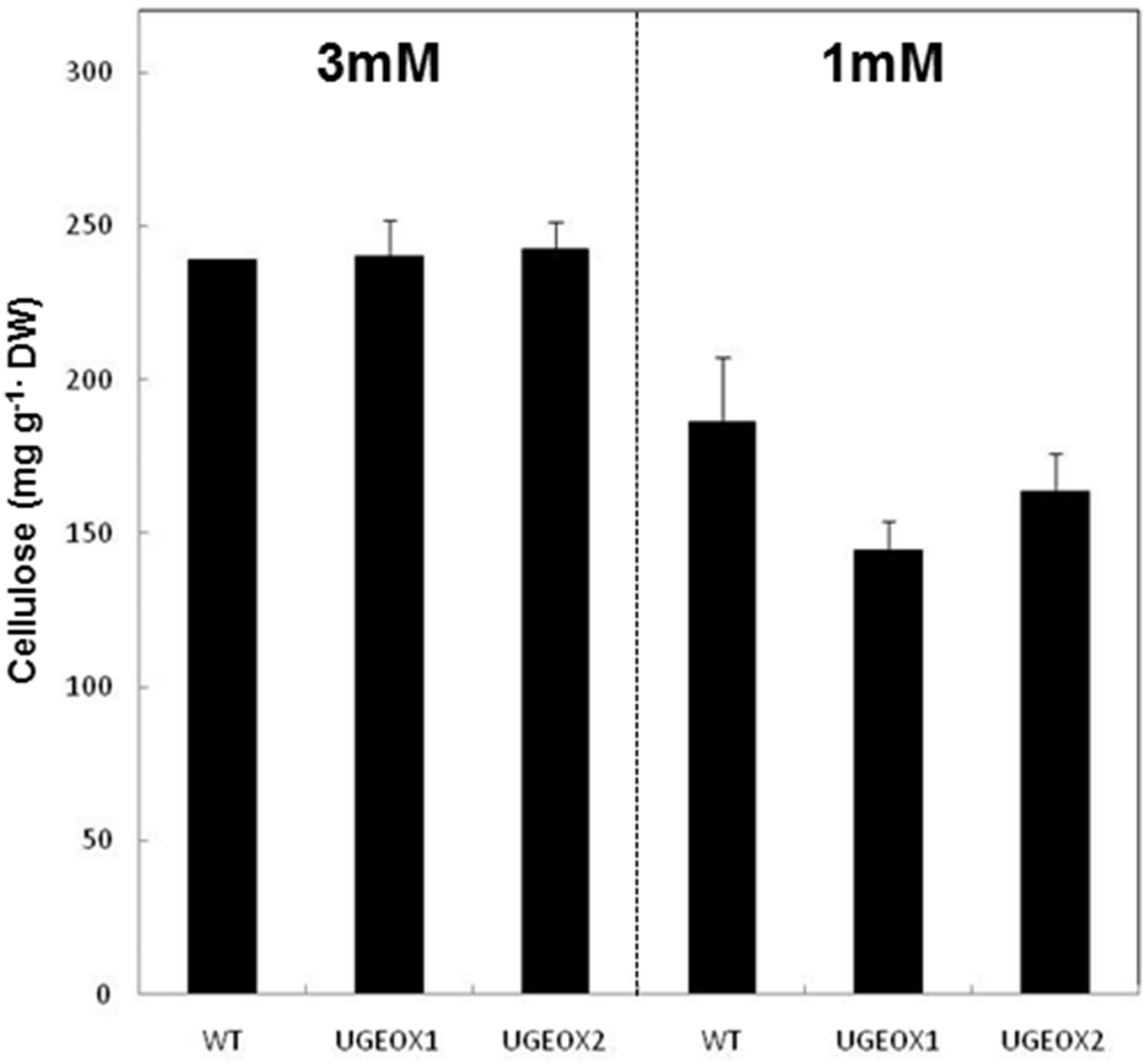
Cellulose content of leaves of hydroponically-grown rice plants subjected to ample (3 mM) or limiting (1 mM) nitrate conditions. Mean±SD, n = 3.

Leaves of wild-type plants subjected to N-limitation contained 22% less cellulose per gram dry weight compared to plants grown under ample N (**Figure 7**). Interestingly, while OsUGE1-OX lines maintained similar amounts of cellulose compared to wild-type plants grown under ample N, OsUGE1-OX lines had 12–22% less cellulose compared to wild-type plants when grown under sub-optimal N-levels (**Figure 7**).

### Expression Profile Analysis of Genes Associated with Cellulose Deposition in Rice

**Figure 8:**
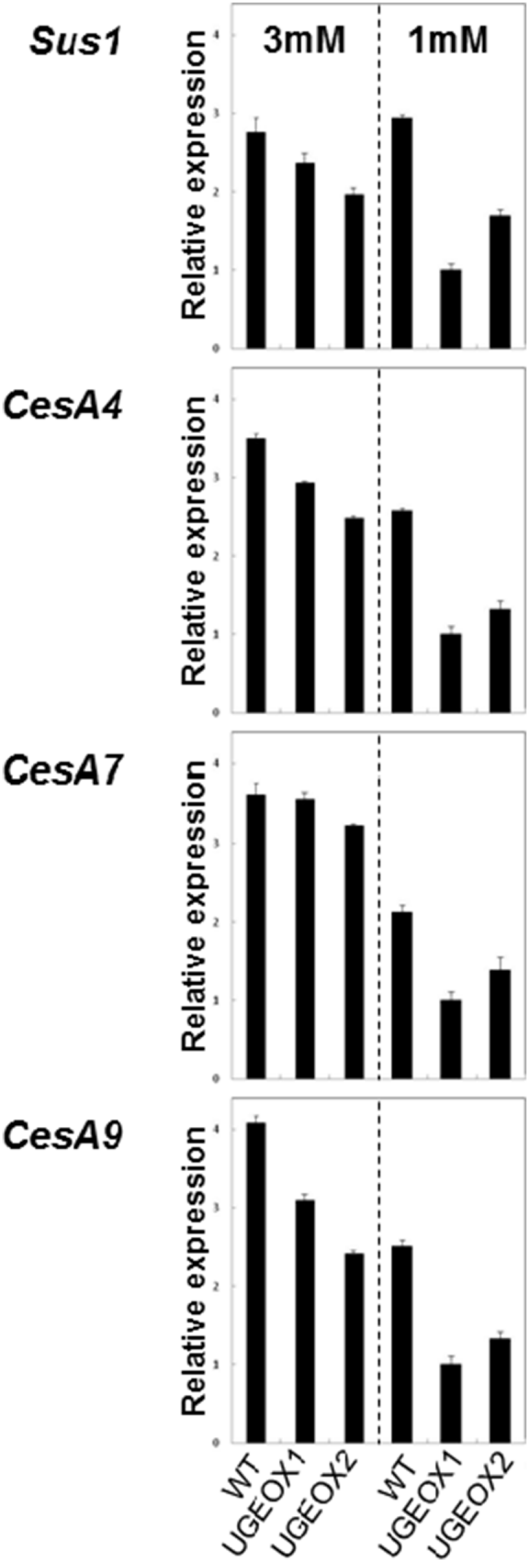
Expression profile of cellulose-associated genes, *Suc1*, *CesA4*, *CesA7*, and *CesA9*, in leaves from hydroponically-grown rice plants subjected to ample (3 mM) or limiting (1 mM) nitrate conditions. Mean±SD, n = 3.

Recent work has shown that cellulose synthesis in plants is accomplished by *cellulose synthase A* (*CesA*) genes which encode products that are arranged in rosette-like structures on the plasma membranes [35], [36]. In rice, *CesA4*, *7*, and *9* have been shown to be indispensable for cellulose biosynthesis [36] and recently, *sucrose synthase 1* (*Sus1*) has been implicated in participating in the provision of UDP-Glc for glucose deposition in the growing cell wall [35]. Given the dramatic decrease in cellulose deposition in leaves of plants subjected to N-limitation (**Figure 7**), we sought to examine whether *Sus1*, and *CesA4*, *7*, and *9* could be implicated in the control of C-flux towards cellulose deposition during N-limiting conditions. The expression of *CesA4*, *7*, and *9* was significantly repressed in wild-type plants subjected to N-limitation compared to ample N, and this response was accentuated in OsUGE1-OX lines (**Figure 8**).

## Discussion

A sub-optimal amount of N has been shown to alter the source:sink balance of plants, leading to a redirection in the diversion of C-skeletons normally used for N assimilation and growth, towards sugar and starch accumulation due to a repression in photosynthesis [3], [4], [12]. However, no studies to date have reported on N-limitation induced changes in rice cell wall polysaccharides. In our study, we monitored both soluble and insoluble carbohydrate responses in rice plants subjected to N-limiting environments, and studied the importance of OsUGE1 in regulating these responses by over-expressing the *OsUGE1* transcription level.

### OsUGE1 Plays an Important Role in Carbohydrate Partitioning to the Cell Wall in Rice

The relative decrease in cellulose content observed in the leaves of rice grown under low N compared to high N suggests that N plays an important role for glucose deposition to polysaccharides of the cell wall during adaptation to N-limitation conditions. In order to understand the mechanism behind this biochemical phenotype, we studied the expression profiles of the sucrose synthase, *OsSus1*, and the cellulose synthase genes *OsCesA 4*, *7* and *9* (**Figure 8**) which have been demonstrated to play critical roles for cellulose deposition in rice [35], [36]. In the wild-type rice plants subjected to N-limitation, *OsCesA 4*, *7*, and *9* were significantly repressed in leaves compared to plants grown under high N (**Figure 8**). Intriguingly, this response was exacerbated in OsUGE1-OX lines subjected to N-limitation (**Figure 8**), which resulted in even lower leaf cellulose contents compared to wild-type plants (**Figure 7**). However, *OsSus1* expression was not affected in plants subjected to N-limitation.

Sucrose synthases catalyze the hydrolysis of sucrose to generate UDP-glc and fructose and have been found to be important for cellulose production by co-localizing and influencing cellulose synthases at the plasma membrane [37]. The repression of *OsSus1* in OsUGE1-OX lines could lead to impaired sucrose synthase-mediated hydrolysis of sucrose and thereby lead to lower UDP-glc availability for cellulose biosynthesis. This could, in part, explain the overall lower cellulosic glucose contents in leaf cell walls on a dry weight basis in OsUGE1-OX lines and higher leaf sucrose contents compared to wild-type plants growth under sub-optimal N-levels.

Recent work has demonstrated the non-redundant functions of multiple UGEs in *Arabidopsis* [17] and barley [27], and multiple UGEs have been found to be necessary to control carbohydrate partitioning [17]. Thus, we cannot rule out the possibility that OsUGE1 and other OsUGEs, together with Sus1, and CesA 4, 7, and 9 might co-ordinate monosaccharide depositions to cell wall polysaccharides. Similar conclusions have been achieved through other previously published works. For example, at least for AtUGEs, it has been shown that all five isoforms display protein-protein interactions with each other [28], and ultimately only AtUGE isoforms 2 and 4 were found to be essential in providing UDP-gal for cell wall biosynthesis in *Arabidopsis* [17]. Furthermore, it has been proposed that cellulose biosynthesis occurs via complex interaction and is not completely understood [38]. Interestingly, we found no evidence for altered hemicellulosic gal or glc in OsUGE1-OX lines compared to wild-type plants grown under high N-treatment. However, OsUGE1-OX lines accumulated proportionately more gal and glc at the expense of xyl in the hemicellulosic polysaccharide profile only during growth under N-limiting conditions, with a concomitant decrease in cell wall cellulose contents (**Figures 6** **,** **7**). Taken together, our findings suggest that OsUGE1 plays a key role for the provision of substrates for cell wall polysaccharide biosynthesis under conditions of lower C-gain that are typified by sub-optimal N environments.

### OsUGE1-OX Coleoptile Growth in the Presence of Exogenous Supplied Gal

In general, similar to Zhu et al. (2009) [34] seeds imbibed in the presence of 55 mM sugar exhibited delayed coleoptile growth and seedling development in rice compared to those that received no sugar (**Figure 5**). Interestingly, OsUGE1-OX coleoptiles were able to grow in the presence of exogenously supplied gal, xyl, or glcA faster than wild-type, and had higher tolerance to galactose (**Figure 5**), a finding consistent with other work reporting that over-expression of specific *UGE*s can help overcome the toxic effects of exogenously applied galactose [19], [39]. Exogenously applied galactose is likely converted to galactose-1-P, and then to UDP-gal, which would be converted to UDP-glc via OsUGE1. Given that UDP-glc is a key substrate for multiple intermediates and end-products of carbohydrate metabolism, its enhanced levels would facilitate the increased biosynthesis of transport sugars such as sucrose, the availability of monosaccharide donors for cell wall polysaccharide biosynthesis in the rapidly expanding coleoptile.

To our surprise, OsUGE1-OX coleoptiles were less inhibited in the presence of xyl or glcA compared to wild-type coleoptiles subjected to identical conditions (**Figure 5**). Furthermore, OsUGE1-OX lines maintained proportionately more gal and glc, and less xyl in the hemicellulosic polysaccharide profile of leaves from plants subjected to N-limitation, compared to wild-type plants. These findings suggest that OsUGE1 participates in the inter-conversion of other sugar nucleotides such as UDP-xyl thereby influencing salvage pathways for sugar nucleotide biosynthesis. Another possibility is that its activity could be modulated in the presence of altered UDP-xyl/UDP-glcA ratios that together influence its ability to interconvert precursors for carbohydrate deposition to both the cellulosic and non-cellulosic components of the cell wall. Work by Kotake et al. (2009) [40] demonstrated that PsUGE1, AtUGE1, and AtUGE3 catalyze interconversions between both UDP-glc and UDP-gal, and UDP-xyl and UDP-ara. It will be important to delineate the physiological importance of the wide substrate specificity for UDP-sugars by UGEs in general, and the mechanism responsible for the diversion of carbon mediated by OsUGE1 during plant adaptation to sub-optimal N conditions.

Three independent *AtUGE1* over-expressing and three antisense transgenic *Arabidopsis* lines revealed no alterations in the morphological and biochemical phenotypes compared to wild-type plants [39]. In this study, over-expression of ***OsUGE1*** showed phenotypic alteration at carbohydrate partitioning level in the transgenic lines, however the idea of producing transgenic lines where ***OsUGE1*** is knocked out/down may show a contrary phenotypic pattern is still valid and would be a future project for this study.

Given the potential of cellulosic biomass for biofuel production, more work is required to understand C-deposition to the cell wall and its genetic control for cell wall polysaccharide composition of crops using biotechnological approaches in order to alter the cell wall structures to generate less rigid forms to increase the efficiency of biofuel production [41]. Our work has demonstrated that one way to alter the cell wall carbohydrate profile is to genetically manipulate the expression of UGEs and grow plants under N-limiting environments that together influence the deposition of carbohydrates in polysaccharide profiles of rice leaves.

## Supporting Information

Figure S1 Sequence analysis of OsUGEs. Alignment of amino acid sequences of rice UGEs. The conserved NAD^+^ binding motif and catalytic amino acid residues of the active site are shown in boxes i and ii, respectively.
(TIF)

Figure S2 The effect of exogenously applied hexoses on dark-grown wild-type rice coleoptiles. Seeds were germinated in the presence of water (control) or 55 mM sugar in the dark, and the coleoptile length was measured after 96 hours. Mean±SD, n = 12, from two separate experiments.
(TIF)

Table S1 Differential metabolic analysis of the UGEO overexpression lines (UGEOX1–2) growing under low (L) and high nitrogen levels (H). Peak areas are normalized to the internal standard, ribitol. Mean ± SD, n = 3.
(DOCX)
