# Supplementary figures and images for: Functional Characterization of the Rice UDP-glucose 4-epimerase 1, OsUGE1: A Potential Role in Cell Wall Carbohydrate Partitioning during Limiting Nitrogen Conditions
Source: PLoS One. 2014 May 1;9(5):e96158. doi: 10.1371/journal.pone.0096158 (PMC4006880; doi:10.1371/journal.pone.0096158)

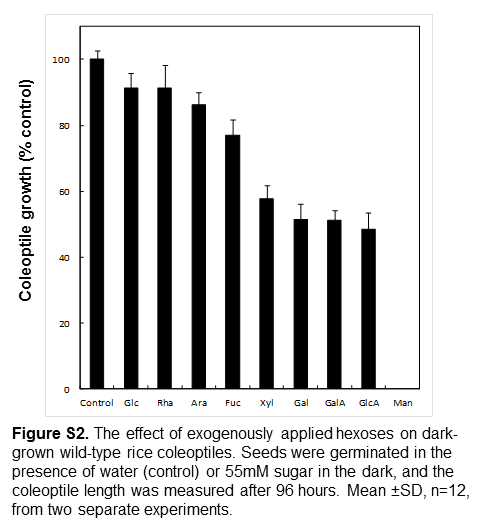

Supplement: Figure S2 — The effect of exogenously applied hexoses on dark-grown wild-type rice coleoptiles. Seeds were germinated in the presence of water (control) or 55 mM sugar in the dark, and the coleoptile length was measured after 96 hours. Mean±SD, n = 12, from two separate experiments. (TIF) [file pone.0096158.s002.tif]
