# Supplementary material for: Functional Characterization of the Rice UDP-glucose 4-epimerase 1, OsUGE1: A Potential Role in Cell Wall Carbohydrate Partitioning during Limiting Nitrogen Conditions
Source: PLoS One. 2014 May 1;9(5):e96158. doi: 10.1371/journal.pone.0096158 (PMC4006880; doi:10.1371/journal.pone.0096158)
Supplement: Table S1 — Differential metabolic analysis of the UGEO overexpression lines (UGEOX1–2) growing under low (L) and high nitrogen levels (H). Peak areas are normalized to the internal standard, ribitol. Mean ± SD, n = 3. (DOCX) [file pone.0096158.s003.docx]

**Table S1.** Differential metabolic analysis of the UGEO overexpression lines (UGEOX1-2) growing under low (L) and high nitrogen levels (H). Peak areas are normalized to the internal standard, ribitol. Mean ± SD, n=3.

| **Metabolite** | **WT-L** | **UGEOX1-L** | ***P-value*** | **UGEOX2-L** | ***P-Value*** | **WT-H** | **UGEOX1-H** | ***P-Value*** | **UGEOX2-H** | ***P-value*** |
| --- | --- | --- | --- | --- | --- | --- | --- | --- | --- | --- |
| **Phosphate** | 1.88±0.65 | 0.80±0.43 | 0.0743 | 0.74±0.17 | 0.0424 | 1.48±0.78 | 1.46±0.68 | 0.9749 | 3.28±1.73 | 0.1758 |
| **Serine** | 0.04±0.02 | 0.03±0.01 | 0.4818 | 0.04±0.02 | 1 | 0.06±0.02 | 0.07±0.04 | 0.7183 | 0.06±0.04 | 1 |
| **Malate** | 0.09±0.02 | 0.12±0.02 | 0.1401 | 0.10±0.03 | 0.656 | 0.20±0.10 | 0.24±0.11 | 0.6654 | 0.40±0.18 | 0.1678 |
| **Glutamate** | 0.10±0.03 | 0.09±0.04 | 0.7465 | 0.12±0.02 | 0.3911 | 0.19±0.07 | 0.36±0.18 | 0.202 | 0.27±0.10 | 0.3197 |
| **Threonine** | 0.20±0.03 | 0.64±0.36 | 0.1025 | 0.48±0.20 | 0.0745 | 0.80±0.31 | 0.88±0.32 | 0.7713 | 1.45±0.71 | 0.2198 |
| **Citrate** | 0.14±0.03 | 0.21±0.07 | 0.1866 | 0.22±0.08 | 0.1802 | 0.33±0.20 | 0.73±0.33 | 0.147 | 0.74±0.52 | 0.2715 |
| **Glutamine** | 0.08±0.04 | 0.10±0.05 | 0.6172 | 0.12±0.03 | 0.2381 | 0.16±0.04 | 0.08±0.06 | 0.127 | 0.16±0.06 | 1 |
| **Quinic acid** | 1.38±0.09 | 2.84±1.32 | 0.1286 | 1.78±0.55 | 0.2817 | 3.14±0.52 | 4.99±1.60 | 0.1295 | 5.41±1.65 | 0.0855 |
| **Fructose** | 0.72±0.06 | 0.87±0.50 | 0.6331 | 0.61±0.28 | 0.5422 | 0.73±0.30 | 1.47±0.80 | 0.208 | 1.11±0.52 | 0.3345 |
| **Galactose** | 0.06±0.02 | 0.08±0.04 | 0.4818 | 0.08±0.03 | 0.3911 | 0.06±0.03 | 0.04±0.01 | 0.3349 | 0.11±0.05 | 0.2117 |
| **Monosaccharide-like unknown** | 0.07±0.02 | 0.10±0.05 | 0.3892 | 0.08±0.03 | 0.656 | 0.07±0.02 | 0.10±0.04 | 0.3099 | 0.11±0.04 | 0.1963 |
| **Myo-inositol** | 0.55±0.12 | 0.46±0.15 | 0.4626 | 0.59±0.15 | 0.7366 | 0.99±0.32 | 1.46±0.80 | 0.3983 | 1.82±0.84 | 0.185 |
| **Glucose-6-Phosphate** | 0.11±0.02 | 0.15±0.03 | 0.127 | 0.14±0.05 | 0.3892 | 0.11±0.04 | 0.37±0.20 | 0.0918 | 0.22±0.11 | 0.1789 |
| **Galactinol** | 0.40±0.03 | 0.65±0.40 | 0.3411 | 0.36±0.07 | 0.4144 | 0.25±0.11 | 0.39±0.17 | 0.2972 | 0.30±0.10 | 0.5915 |
| **Raffinose** | 0.28±0.12 | 0.50±0.19 | 0.1652 | 0.24±0.16 | 0.7465 | 0.40±0.18 | 0.36±0.11 | 0.7591 | 0.67±0.23 | 0.1846 |
